# Supplementary material for: OpenMM 7: Rapid development of high performance algorithms for molecular dynamics
Source: PLoS Comput Biol. 2017 Jul 26;13(7):e1005659. doi: 10.1371/journal.pcbi.1005659 (PMC5549999; doi:10.1371/journal.pcbi.1005659)
Supplement: S1 Source Code — (ZIP) [file pcbi.1005659.s002.zip › OpenMM-7.0.1-Source/docs/api-c++/generated/OpenMM.AmoebaMultipoleForce.html]

AmoebaMultipoleForce — OpenMM @OPENMM\_MAJOR\_VERSION@.@OPENMM\_MINOR\_VERSION@ documentation


# AmoebaMultipoleForce¶

*class* `OpenMM::``AmoebaMultipoleForce`¶
:   This class implements the Amoeba multipole interaction.

    To use it, create an `AmoebaMultipoleForce` object then call `addMultipole()` once for each atom. After an entry has been added, you can modify its force field parameters by calling `setMultipoleParameters()`. This will have no effect on Contexts that already exist unless you call `updateParametersInContext()`.

    Methods

    |  |  |
    | --- | --- |
    | `AmoebaMultipoleForce` | Create an `AmoebaMultipoleForce`. |
    | `getNumMultipoles` | Get the number of particles in the potential function |
    | `getNonbondedMethod` | Get the method used for handling long-range nonbonded interactions. |
    | `setNonbondedMethod` | Set the method used for handling long-range nonbonded interactions. |
    | `getPolarizationType` | Get polarization type |
    | `setPolarizationType` | Set the polarization type |
    | `getCutoffDistance` | Get the cutoff distance (in nm) being used for nonbonded interactions. |
    | `setCutoffDistance` | Set the cutoff distance (in nm) being used for nonbonded interactions. |
    | `getAEwald` | Get the Ewald alpha parameter. |
    | `setAEwald` | Set the Ewald alpha parameter. |
    | `getPmeBSplineOrder` | Get the B-spline order to use for PME charge spreading |
    | `getPmeGridDimensions` | Get the PME grid dimensions. |
    | `setPmeGridDimensions` | Set the PME grid dimensions. |
    | `getPMEParametersInContext` | Get the parameters being used for PME in a particular `Context`. |
    | `addMultipole` | Add multipole-related info for a particle |
    | `getMultipoleParameters` | Get the multipole parameters for a particle. |
    | `setMultipoleParameters` | Set the multipole parameters for a particle. |
    | `setCovalentMap` | Set the CovalentMap for an atom |
    | `getCovalentMap` | Get the CovalentMap for an atom |
    | `getCovalentMaps` | Get the CovalentMap for an atom |
    | `getMutualInducedMaxIterations` | Get the max number of iterations to be used in calculating the mutual induced dipoles |
    | `setMutualInducedMaxIterations` | Set the max number of iterations to be used in calculating the mutual induced dipoles |
    | `getMutualInducedTargetEpsilon` | Get the target epsilon to be used to test for convergence of iterative method used in calculating the mutual induced dipoles |
    | `setMutualInducedTargetEpsilon` | Set the target epsilon to be used to test for convergence of iterative method used in calculating the mutual induced dipoles |
    | `setExtrapolationCoefficients` | Set the coefficients for the mu\_0, mu\_1, mu\_2, ..., mu\_n terms in the extrapolation algorithm for induced dipoles. |
    | `getExtrapolationCoefficients` | Get the coefficients for the mu\_0, mu\_1, mu\_2, ..., mu\_n terms in the extrapolation algorithm for induced dipoles. |
    | `getEwaldErrorTolerance` | Get the error tolerance for Ewald summation. |
    | `setEwaldErrorTolerance` | Get the error tolerance for Ewald summation. |
    | `getInducedDipoles` | Get the induced dipole moments of all particles. |
    | `getElectrostaticPotential` | Get the electrostatic potential. |
    | `getSystemMultipoleMoments` | Get the system multipole moments. |
    | `updateParametersInContext` | Update the multipole parameters in a `Context` to match those stored in this `Force` object. |
    | `usesPeriodicBoundaryConditions` | Returns whether or not this force makes use of periodic boundary conditions. |

    Enum: NonbondedMethod

    |  |  |
    | --- | --- |
    | **NoCutoff** | No cutoff is applied to nonbonded interactions. The full set of N^2 interactions is computed exactly. This necessarily means that periodic boundary conditions cannot be used. This is the default. |
    | **PME** | Periodic boundary conditions are used, and Particle-Mesh Ewald (PME) summation is used to compute the interaction of each particle with all periodic copies of every other particle. |

    Enum: PolarizationType

    |  |  |
    | --- | --- |
    | **Mutual** | Full mutually induced polarization. The dipoles are iterated until the converge to the accuracy specified by `getMutualInducedTargetEpsilon()`. |
    | **Direct** | Direct polarization approximation. The induced dipoles depend only on the fixed multipoles, not on other induced dipoles. |
    | **Extrapolated** | Extrapolated perturbation theory approximation. The dipoles are iterated a few times, and then an analytic approximation is used to extrapolate to the fully converged values. Call `setExtrapolationCoefficients()` to set the coefficients used for the extrapolation. The default coefficients used in this release are [-0.154, 0.017, 0.658, 0.474], but be aware that those may change in a future release. |

    Enum: MultipoleAxisTypes

    |  |  |
    | --- | --- |
    | **ZThenX** |  |
    | **Bisector** |  |
    | **ZBisect** |  |
    | **ThreeFold** |  |
    | **ZOnly** |  |
    | **NoAxisType** |  |
    | **LastAxisTypeIndex** |  |

    Enum: CovalentType

    |  |  |
    | --- | --- |
    | **Covalent12** |  |
    | **Covalent13** |  |
    | **Covalent14** |  |
    | **Covalent15** |  |
    | **PolarizationCovalent11** |  |
    | **PolarizationCovalent12** |  |
    | **PolarizationCovalent13** |  |
    | **PolarizationCovalent14** |  |
    | **CovalentEnd** |  |

    `AmoebaMultipoleForce`()¶
    :   Create an `AmoebaMultipoleForce`.

    int `getNumMultipoles`() *const*¶
    :   Get the number of particles in the potential function

    NonbondedMethod `getNonbondedMethod`() *const*¶
    :   Get the method used for handling long-range nonbonded interactions.

    void `setNonbondedMethod`(NonbondedMethod *method*)¶
    :   Set the method used for handling long-range nonbonded interactions.

    PolarizationType `getPolarizationType`() *const*¶
    :   Get polarization type

    void `setPolarizationType`(PolarizationType *type*)¶
    :   Set the polarization type

    double `getCutoffDistance`() *const*¶
    :   Get the cutoff distance (in nm) being used for nonbonded interactions. If the NonbondedMethod in use is NoCutoff, this value will have no effect.

        |  |  |
        | --- | --- |
        | Returns: | the cutoff distance, measured in nm |

    void `setCutoffDistance`(double *distance*)¶
    :   Set the cutoff distance (in nm) being used for nonbonded interactions. If the NonbondedMethod in use is NoCutoff, this value will have no effect.

        |  |  |
        | --- | --- |
        | Parameters: |  |

        - **distance** – the cutoff distance, measured in nm

    double `getAEwald`() *const*¶
    :   Get the Ewald alpha parameter. If this is 0 (the default), a value is chosen automatically based on the Ewald error tolerance.

        |  |  |
        | --- | --- |
        | Returns: | the Ewald alpha parameter |

    void `setAEwald`(double *aewald*)¶
    :   Set the Ewald alpha parameter. If this is 0 (the default), a value is chosen automatically based on the Ewald error tolerance.

        |  |  |
        | --- | --- |
        | Parameters: |  |

        - **aewald** – alpha parameter

    int `getPmeBSplineOrder`() *const*¶
    :   Get the B-spline order to use for PME charge spreading

        |  |  |
        | --- | --- |
        | Returns: | the B-spline order |

    void `getPmeGridDimensions`(std::vector<int> &*gridDimension*) *const*¶
    :   Get the PME grid dimensions. If Ewald alpha is 0 (the default), this is ignored and grid dimensions are chosen automatically based on the Ewald error tolerance.

        |  |  |
        | --- | --- |
        | Returns: | the PME grid dimensions |

    void `setPmeGridDimensions`(*const* std::vector<int> &*gridDimension*)¶
    :   Set the PME grid dimensions. If Ewald alpha is 0 (the default), this is ignored and grid dimensions are chosen automatically based on the Ewald error tolerance.

        |  |  |
        | --- | --- |
        | Parameters: |  |

        - **gridDimension** – the PME grid dimensions

    void `getPMEParametersInContext`(*const* Context &*context*, double &*alpha*, int &*nx*, int &*ny*, int &*nz*) *const*¶
    :   Get the parameters being used for PME in a particular `Context`. Because some platforms have restrictions on the allowed grid sizes, the values that are actually used may be slightly different from those specified with `setPmeGridDimensions()`, or the standard values calculated based on the Ewald error tolerance. See the manual for details.

        |  |  |
        | --- | --- |
        | Parameters: |  |

        - **context** – the `Context` for which to get the parameters
        - **alpha** – [out] the separation parameter
        - **nx** – [out] the number of grid points along the X axis
        - **ny** – [out] the number of grid points along the Y axis
        - **nz** – [out] the number of grid points along the Z axis

    int `addMultipole`(double *charge*, *const* std::vector<double> &*molecularDipole*, *const* std::vector<double> &*molecularQuadrupole*, int *axisType*, int *multipoleAtomZ*, int *multipoleAtomX*, int *multipoleAtomY*, double *thole*, double *dampingFactor*, double *polarity*)¶
    :   Add multipole-related info for a particle

        |  |  |
        | --- | --- |
        | Parameters: |  |

        - **charge** – the particle’s charge
        - **molecularDipole** – the particle’s molecular dipole (vector of size 3)
        - **molecularQuadrupole** – the particle’s molecular quadrupole (vector of size 9)
        - **axisType** – the particle’s axis type
        - **multipoleAtomZ** – index of first atom used in constructing lab<->molecular frames
        - **multipoleAtomX** – index of second atom used in constructing lab<->molecular frames
        - **multipoleAtomY** – index of second atom used in constructing lab<->molecular frames
        - **thole** – Thole parameter
        - **dampingFactor** – dampingFactor parameter
        - **polarity** – polarity parameter

        |  |  |
        | --- | --- |
        | Returns: | the index of the particle that was added |

    void `getMultipoleParameters`(int *index*, double &*charge*, std::vector<double> &*molecularDipole*, std::vector<double> &*molecularQuadrupole*, int &*axisType*, int &*multipoleAtomZ*, int &*multipoleAtomX*, int &*multipoleAtomY*, double &*thole*, double &*dampingFactor*, double &*polarity*) *const*¶
    :   Get the multipole parameters for a particle.

        |  |  |
        | --- | --- |
        | Parameters: |  |

        - **index** – the index of the atom for which to get parameters
        - **charge** – [out] the particle’s charge
        - **molecularDipole** – [out] the particle’s molecular dipole (vector of size 3)
        - **molecularQuadrupole** – [out] the particle’s molecular quadrupole (vector of size 9)
        - **axisType** – [out] the particle’s axis type
        - **multipoleAtomZ** – [out] index of first atom used in constructing lab<->molecular frames
        - **multipoleAtomX** – [out] index of second atom used in constructing lab<->molecular frames
        - **multipoleAtomY** – [out] index of second atom used in constructing lab<->molecular frames
        - **thole** – [out] Thole parameter
        - **dampingFactor** – [out] dampingFactor parameter
        - **polarity** – [out] polarity parameter

    void `setMultipoleParameters`(int *index*, double *charge*, *const* std::vector<double> &*molecularDipole*, *const* std::vector<double> &*molecularQuadrupole*, int *axisType*, int *multipoleAtomZ*, int *multipoleAtomX*, int *multipoleAtomY*, double *thole*, double *dampingFactor*, double *polarity*)¶
    :   Set the multipole parameters for a particle.

        |  |  |
        | --- | --- |
        | Parameters: |  |

        - **index** – the index of the atom for which to set parameters
        - **charge** – the particle’s charge
        - **molecularDipole** – the particle’s molecular dipole (vector of size 3)
        - **molecularQuadrupole** – the particle’s molecular quadrupole (vector of size 9)
        - **axisType** – the particle’s axis type
        - **multipoleAtomZ** – index of first atom used in constructing lab<->molecular frames
        - **multipoleAtomX** – index of second atom used in constructing lab<->molecular frames
        - **multipoleAtomY** – index of second atom used in constructing lab<->molecular frames
        - **thole** – thole parameter
        - **dampingFactor** – damping factor parameter
        - **polarity** – polarity parameter

    void `setCovalentMap`(int *index*, CovalentType *typeId*, *const* std::vector<int> &*covalentAtoms*)¶
    :   Set the CovalentMap for an atom

        |  |  |
        | --- | --- |
        | Parameters: |  |

        - **index** – the index of the atom for which to set parameters
        - **typeId** – CovalentTypes type
        - **covalentAtoms** – vector of covalent atoms associated w/ the specfied CovalentType

    void `getCovalentMap`(int *index*, CovalentType *typeId*, std::vector<int> &*covalentAtoms*) *const*¶
    :   Get the CovalentMap for an atom

        |  |  |
        | --- | --- |
        | Parameters: |  |

        - **index** – the index of the atom for which to set parameters
        - **typeId** – CovalentTypes type
        - **covalentAtoms** – [out] output vector of covalent atoms associated w/ the specfied CovalentType

    void `getCovalentMaps`(int *index*, std::vector<std::vector<int>> &*covalentLists*) *const*¶
    :   Get the CovalentMap for an atom

        |  |  |
        | --- | --- |
        | Parameters: |  |

        - **index** – the index of the atom for which to set parameters
        - **covalentLists** – [out] output vector of covalent lists of atoms

    int `getMutualInducedMaxIterations`(void) *const*¶
    :   Get the max number of iterations to be used in calculating the mutual induced dipoles

        |  |  |
        | --- | --- |
        | Returns: | max number of iterations |

    void `setMutualInducedMaxIterations`(int *inputMutualInducedMaxIterations*)¶
    :   Set the max number of iterations to be used in calculating the mutual induced dipoles

        |  |  |
        | --- | --- |
        | Parameters: |  |

        - **inputMutualInducedMaxIterations** – number of iterations

    double `getMutualInducedTargetEpsilon`(void) *const*¶
    :   Get the target epsilon to be used to test for convergence of iterative method used in calculating the mutual induced dipoles

        |  |  |
        | --- | --- |
        | Returns: | target epsilon |

    void `setMutualInducedTargetEpsilon`(double *inputMutualInducedTargetEpsilon*)¶
    :   Set the target epsilon to be used to test for convergence of iterative method used in calculating the mutual induced dipoles

        |  |  |
        | --- | --- |
        | Parameters: |  |

        - **inputMutualInducedTargetEpsilon** – target epsilon

    void `setExtrapolationCoefficients`(*const* std::vector<double> &*coefficients*)¶
    :   Set the coefficients for the mu\_0, mu\_1, mu\_2, ..., mu\_n terms in the extrapolation algorithm for induced dipoles.

        |  |  |
        | --- | --- |
        | Parameters: |  |

        - **coefficients** – a vector whose mth entry specifies the coefficient for mu\_m. The length of this vector determines how many iterations are performed.

    *const* std::vector<double> &`getExtrapolationCoefficients`() *const*¶
    :   Get the coefficients for the mu\_0, mu\_1, mu\_2, ..., mu\_n terms in the extrapolation algorithm for induced dipoles. In this release, the default values for the coefficients are [-0.154, 0.017, 0.658, 0.474], but be aware that those may change in a future release.

    double `getEwaldErrorTolerance`() *const*¶
    :   Get the error tolerance for Ewald summation. This corresponds to the fractional error in the forces which is acceptable. This value is used to select the grid dimensions and separation (alpha) parameter so that the average error level will be less than the tolerance. There is not a rigorous guarantee that all forces on all atoms will be less than the tolerance, however.

        This can be overridden by explicitly setting an alpha parameter and grid dimensions to use.

    void `setEwaldErrorTolerance`(double *tol*)¶
    :   Get the error tolerance for Ewald summation. This corresponds to the fractional error in the forces which is acceptable. This value is used to select the grid dimensions and separation (alpha) parameter so that the average error level will be less than the tolerance. There is not a rigorous guarantee that all forces on all atoms will be less than the tolerance, however.

        This can be overridden by explicitly setting an alpha parameter and grid dimensions to use.

    void `getInducedDipoles`(Context &*context*, std::vector<Vec3> &*dipoles*)¶
    :   Get the induced dipole moments of all particles.

        |  |  |
        | --- | --- |
        | Parameters: |  |

        - **context** – the `Context` for which to get the induced dipoles
        - **dipoles** – [out] the induced dipole moment of particle i is stored into the i’th element

    void `getElectrostaticPotential`(*const* std::vector<Vec3> &*inputGrid*, Context &*context*, std::vector<double> &*outputElectrostaticPotential*)¶
    :   Get the electrostatic potential.

        |  |  |
        | --- | --- |
        | Parameters: |  |

        - **inputGrid** – input grid points over which the potential is to be evaluated
        - **context** – context
        - **outputElectrostaticPotential** – [out] output potential

    void `getSystemMultipoleMoments`(Context &*context*, std::vector<double> &*outputMultipoleMoments*)¶
    :   Get the system multipole moments.

        This method is most useful for non-periodic systems. When called for a periodic system, only the

        |  |  |
        | --- | --- |
        | Parameters: |  |

        - **context** – context
        - **outputMultipoleMoments** – [out] (charge, dipole\_x, dipole\_y, dipole\_z, quadrupole\_xx, quadrupole\_xy, quadrupole\_xz, quadrupole\_yx, quadrupole\_yy, quadrupole\_yz, quadrupole\_zx, quadrupole\_zy, quadrupole\_zz)

    void `updateParametersInContext`(Context &*context*)¶
    :   Update the multipole parameters in a `Context` to match those stored in this `Force` object. This method provides an efficient method to update certain parameters in an existing `Context` without needing to reinitialize it. Simply call `setMultipoleParameters()` to modify this object’s parameters, then call `updateParametersInContext()` to copy them over to the `Context`.

        This method has several limitations. The only information it updates is the parameters of multipoles. All other aspects of the `Force` (the nonbonded method, the cutoff distance, etc.) are unaffected and can only be changed by reinitializing the `Context`. Furthermore, this method cannot be used to add new multipoles, only to change the parameters of existing ones.

    bool `usesPeriodicBoundaryConditions`() *const*¶
    :   Returns whether or not this force makes use of periodic boundary conditions.

        |  |  |
        | --- | --- |
        | Returns: | true if nonbondedMethod uses PBC and false otherwise |

High performance molecular simulation on GPUs


### Navigation

- Core Objects
- Forces
- Integrators
- Extras

©2015, Stanford University and the Authors.
|
Powered by Sphinx 1.3.1
& Alabaster 0.7.7
|
Page source
